# Supplementary material for: Clinical Outcomes of Patients with Chronic Neuropathic Form of Gaucher Disease in the Spanish Real-World Setting: A Retrospective Study
Source: Biomedicines. 2023 Oct 22;11(10):2861. doi: 10.3390/biomedicines11102861 (PMC10603893; doi:10.3390/biomedicines11102861)
Supplement: Supplementary file 1 [file biomedicines-11-02861-s001.zip › biomedicines-2651402-supplementary.pdf]

Supplementary table 1. Demographic data.

Patient code	Age at inclusion (years)	Age at disease onset (years)	Age at diagnosis (years)	Sex
1	9	0.9	0.9	Male
2	15	3	3	Male
3	26	0.2	0.2	Male
4	22	20	20	Male
5	4	1.1	1.2	Female
6	8	2	2	Male
7	21	0.03	0.5	Male
8	26	1.5	1.5	Male
9	21	1	1	Male
10	16	0.9	0.9	Male
11	12	1.7	1.9	Female
12	16	0.8	1	Male
13	10	2.5	3.5	Female
14	8	1	1.2	Male
15	8	1	1.2	Female
16	1	0.6	1.1	Female
17	15	1	1.5	Male
18	24	0	0	Male
19	5	0.42	0.58	Male

Supplementary table 2. Medical history.

Medical history	Status	n	%
**Normal pregnancy**	Yes	14	74
	No	4	21
	Unknown	1	5
**Normal perinatal development**	Yes	5	26
	No	13	68
	Unknown	1	5
**Family history of GD3**	Yes	3	16
	No	15	79
	Unknown	1	5
**Dementias**	Yes	0	0
	No	18	95
	Unknown	1	5
**Familial Parkinson**	Yes	1	5
	No	17	89
	Unknown	1	5
**Consanguinity**	Yes	4	21
	No	15	79
	Unknown	0	0
